# Deciphering the conserved genetic loci implicated in plant disease control through comparative genomics of *Bacillus amyloliquefaciens* subsp. *plantarum*

**DOI:** 10.3389/fpls.2015.00631

**Published:** 2015-08-17

**Authors:** Mohammad J. Hossain, Chao Ran, Ke Liu, Choong-Min Ryu, Cody R. Rasmussen-Ivey, Malachi A. Williams, Mohammad K. Hassan, Soo-Keun Choi, Haeyoung Jeong, Molli Newman, Joseph W. Kloepper, Mark R. Liles

**Affiliations:** ^1^Department of Biological Sciences, Auburn UniversityAuburn, AL, USA; ^2^Department of Entomology and Plant Pathology, Auburn UniversityAuburn, AL, USA; ^3^Superbacteria Research Center, Korea Research Institute of Bioscience & BiotechnologyDaejeon, South Korea

**Keywords:** *Bacillus*, *plantarum*, host colonization, biocontrol, bacterial spot disease, PGPR

## Abstract

To understand the growth-promoting and disease-inhibiting activities of plant growth-promoting rhizobacteria (PGPR) strains, the genomes of 12 *Bacillus subtilis* group strains with PGPR activity were sequenced and analyzed. These *B. subtilis* strains exhibited high genomic diversity, whereas the genomes of *B. amyloliquefaciens* strains (a member of the *B. subtilis* group) are highly conserved. A pairwise BLASTp matrix revealed that gene family similarity among *Bacillus* genomes ranges from 32 to 90%, with 2839 genes within the core genome of *B. amyloliquefaciens* subsp. *plantarum*. Comparative genomic analyses of *B. amyloliquefaciens* strains identified genes that are linked with biological control and colonization of roots and/or leaves, including 73 genes uniquely associated with subsp. *plantarum* strains that have predicted functions related to signaling, transportation, secondary metabolite production, and carbon source utilization. Although *B. amyloliquefaciens* subsp. *plantarum* strains contain gene clusters that encode many different secondary metabolites, only polyketide biosynthetic clusters that encode difficidin and macrolactin are conserved within this subspecies. To evaluate their role in plant pathogen biocontrol, genes involved in secondary metabolite biosynthesis were deleted in a *B. amyloliquefaciens* subsp. *plantarum* strain, revealing that difficidin expression is critical in reducing the severity of disease, caused by *Xanthomonas axonopodis* pv. vesicatoria in tomato plants. This study defines genomic features of PGPR strains and links them with biocontrol activity and with host colonization.

## Introduction

Bacteria associated with plant roots that exert beneficial effects on plant growth and development are referred to as plant growth–promoting rhizobacteria (PGPR) (Kloepper and Schroth, 1978; Kloepper et al., 2004). *Bacillus* and *Pseudomonas* spp. are predominant among the diverse bacterial genera that have been linked with PGPR activity (Podile and Kishore, 2006). Members of the *B. subtilis* group, including *B. subtilis, B. licheniformis, B. pumilus, B. amyloliquefaciens, B. atrophaeus, B. mojavensis, B. vallismortis, B. sonorensis*, and *B. tequilensis* have been identified as PGPR strains for their capacity to stimulate plant growth and suppress pathogens within rhizosphere and phyllosphere (Kloepper et al., 2004; Hao et al., 2012; Kim et al., 2012). Strains of *B. amyloliquefaciens* are widely used for their positive effects on plant growth (Idriss et al., 2002). Reva et al. (2004) reported that seven *Bacillus* isolates from plants or soil are closely related yet distinct from *B. amyloliquefaciens* type strain DSM7^T^. In addition, these strains are more proficient for rhizosphere colonization than other members of the *B. subtilis* group. GB03 (Nakkeeran et al., 2005), INR7 (Kokalis–Burelle et al., 2002), and FZB42 (Chen et al., 2007) are PGPR strains within the *Bacillus subtilis* group that have been widely used in different commercial formulations to promote plant growth.

In addition to promoting plant growth, PGPR strains may exhibit biological control of plant diseases. Antibiosis, through the production of inhibitory bioactive compounds, and induced systemic resistance are widely reported biological control mechanisms of *Bacillus* spp. PGPR strains (Ryu et al., 2004). PGPR *Bacillus* spp. strains produce diverse antimicrobial compounds including antibiotics (Emmert et al., 2004), volatile organic compounds (VOCs) (Yuan et al., 2012), and lipopeptides (Ongena et al., 2007) that are associated with the observed biocontrol activity against plant pathogens. For example, *B. amyloliquefaciens* NJN-6 produces 11 VOCs that provide antifungal activity against *Fusarium oxysporum* f. sp. *cubense* (Yuan et al., 2012). Similarly, *B. subtils* strains produce lipopeptides (e.g., surfactin and fengycin), that induce systemic resistance in bean plants (Ongena et al., 2007).

PGPR strains usually need to colonize plant roots extensively to exert plant growth promoting effects using both direct and indirect mechanisms (Lugtenberg and Kamilova, 2009), extensive root colonization is not required for induced systemic resistance (ISR) (Kamilova et al., 2005). In some PGPR strains, root colonization is a prerequisite for biocontrol activity through antibiosis (Chin et al., 2000). For example, *B. amyloliquefaciens* subsp. *plantarum* FZB42 exerts growth promoting activities through efficient colonization of plant roots (Fan et al., 2011). Previously, it has been demonstrated that over-expression of genes involved in phosphorylation of DegU, a two-component response regulator of *B. amyloliquefaciens* strain SQR9, positively influences root colonization as well as other growth-promoting activities by PGPR strains for controlling cucumber wilt disease (Xu et al., 2014). Moreover, the root colonization capacity of a poor root colonizer can be improved by cloning genes that are required for efficient root colonization (Dekkers et al., 2000). Competitive root colonization by PGPR are controlled by many genes and/or genetic cluster(s) (Dietel et al., 2013), so identification of these genetic loci involved in competitive root colonization are challenging if genome sequences are lacking for those PGPR strains (Lugtenberg and Kamilova, 2009). Analysis of additional PGPR strains will help elucidate the mechanisms of competitive root colonization, antibiosis and ISR of PGPR strains and form a foundation for genetic engineering and other strategies to increase the plant-growth promoting capacity of these bacteria.

In this study, we sequenced the genomes of 12 *Bacillus subtilis* group isolates from diverse locales. Comparative genomic analyses of PGPR strains and control strains of the *B. subtilis* group without any reported biocontrol activity against plant pathogens provides insight into genomic features involved in PGPR activity. PGPR strain AP193, which inhibits growth of plant and animal bacterial pathogens (Ran et al., 2012), is an ideal candidate to evaluate the relative contribution of genes that are predicted to be involved in the biosynthesis of bioactive secondary metabolites that could contribute to biocontrol activity, specifically difficidin (*dfnD* mutant), surfactin (*srfAA* mutant), as well as all polyketides and lipopeptides produced by non-ribosomal peptide synthesis, including difficidin (*sfp* mutant). Mutants were then tested for their ability to inhibit plant pathogens *in vitro* and control bacterial spot disease in tomato.

## Materials and methods

### Bacterial strains, plasmids, and growth conditions

Bacterial strains and plasmids used in this study are listed in Table 1. *E. coli* and *Bacillus* strains were grown in Luria-Bertani (LB) medium; however, for electrocompetent cell preparation, *Bacillus amyloliquefaciens* subsp. *plantarum* AP193 was grown in NCM medium (17.4 g K_2_HPO_4_, 11.6 g NaCl, 5 g glucose, 5 g tryptone, 1 g yeast extract, 0.3 g trisodium citrate, 0.05 g MgSO_4_·7H_2_O and 91.1 g sorbitol in 1 L deionized water, pH 7.2). For production of secondary metabolites, *Bacillus* cultures were grown for 48 h at 30°C in Tryptic Soy broth (TSB). In addition, ampicillin (100 μg/ml), chloramphenicol (12.5 μg/ml), or erythromycin (200 μg/ml for *E. coli* or 5 μg/ml for *Bacillus*) were used as selective agents in growth media as required.

**Table 1:** **Bacterial strains and plasmids used in this study**.

**Strains or plasmids**	**Relevant characteristics**	**Source or reference**
*E. coli* K12 ER2925	*dcm-6 dam13::Tn9*	New England Biolabs
*B. amyloliquefaciens* subsp. plantarum strain AP193	Wild type	Dr. Joseph Kloepper (Department of Entomology and Plant Pathology, Auburn University)
AP193Δ*sfp*	Deficient in lipopeptides and polyketides	This study
AP193Δ*srfAA*	Deficient in surfactin production	This study
AP193Δ*dfnD*	Deficient in difficidin production	This study
*Bacillus amyloliquefaciens* FZB42	Wild type	Chen et al., 2007
pMK4	*E. coli*-*Bacillus* shuttle plasmid, rolling circle replicative, Cm^R^	BGSC
pNZT1	Replication thermosensitive derivative of the rolling-circle plasmid pWV01 (pG^+^ replicon, Em^R^)	Xiaozhou Zhang, Virginia Tech
pNZ-sfp	pNZT1 with upstream and downstream sequences of gene *sfp*	This study
pNZ-srf	pNZT1 with knock-out construct of *srfAA*	This study
pNZ-dif	pNZT1 with knock-out construct of *dfn*D	This study

### Sequencing, assembly and annotation

Next-generation sequencing of *Bacillus* spp. genomes was performed using Illumina and Roche 454 sequencing platforms. Indexed Illumina libraries were prepared for strains AP71, AP79, and AB01 using Nextera DNA Sample Prep Kit (Epicentre, Madison, WI) and sequences were generated using an Illumina MiSeq with a 2 × 250 paired end sequencing kit. Barcoded Illumina libraries for strains AP143, AP193, and AP254 were constructed using a NxSeq® DNA Sample Prep Kit (Lucigen, Middleton, WI) and sequenced at EnGenCore (Univ. of South Carolina) using the 454-pyrosequencing platform. Genomic DNA library construction and sequencing for *Bacillus subtilis* GB03, *Bacillus pumilus* INR7, *B. mojavensis* KCTC 3706T, *B. tequilensis* KCTC 13622T, *Bacillus siamensis* KCTC 13613T, and *B. sonorensis* KCTC 13918T were conducted at the National Instrument Center for Environmental Management (Seoul, Republic of Korea), using the Illumina HiSeq 2000 sequencing platform. Sequence reads were trimmed for quality then assembled *de novo* using the CLC Genomics Workbench (CLCBio, Cambridge, MA). Gene prediction and annotation were performed using GeneMark (Lukashin and Borodovsky, 1998) and the RAST annotation server (Aziz et al., 2008), respectively. The identity of individual open reading frames (ORFs) from secondary metabolite biosynthesis gene clusters was confirmed by BLASTx against the GenBank database. Genome sequence reads for strains AB01, AP71, AP79, AP143, AP193, AP254, GB03 (Choi et al., 2014), INR7 (Jeong et al., 2014), KCTC 3706T, KCTC 13613T (Jeong et al., 2012), KCTC 13918T, and KCTC 13622T were deposited into the Short Read Archive (SRA) at NCBI under the accession numbers SRR1176001, SRR1176002, SRR1176003, SRR1176004, SRR1176085, and SRR1176086, SRR1034787, SRR1141652, SRR1141654, SRR1144835, SRR1144836, and SRR1144837, respectively.

### Determination of average nucleotide identity

Average nucleotide identities (ANI) between genomes were calculated using an ANI calculator that estimates ANI according to the methods described previously (Goris et al., 2007).

### Phylogenetic analysis of bacillus species

**Figure 1:**
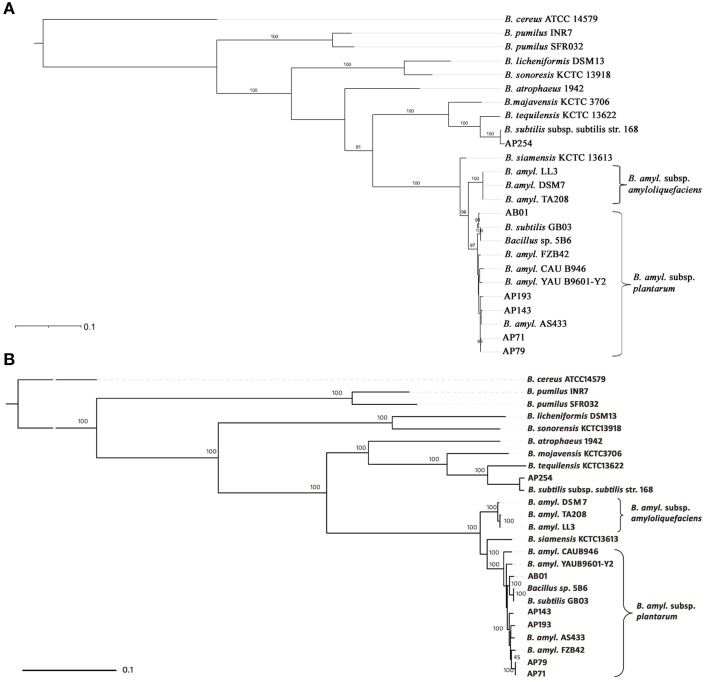
**Phylogeny of PGPR ***Bacillus*** spp. evaluated in this study. (A)** Neighbor joining phylogenetic tree based on *gyrB* sequences using *B. cereus* ATCC 14579^*T*^ as an outgroup. **(B)** Maximum-likelihood phylogenetic tree of the 25 *B. subtilis* group strains based on 729,383 bp sequence of core genome. Two clusters belonging to *B. amyloliquefaciens* subsp. *plantarum* and *B. amyloliquefaciens* subsp. *amyloliquefaciens* are indicated by brackets.

For phylogenetic analysis, the *gyrB* gene sequence for each strain (a list of the 25 strains is presented in Figure 1) was retrieved from sequence data. Strains AS43.3, FZB42, YAU B9601-Y2, CAU B946, and 5B6 were used as representative strains of *B. amyloliquefaciens* subsp. *plantarum*; strains DSM7, LL3, and TA208 were used as representative strains of *B. amyloliquefaciens* subsp. *amyloliquefaciens*. The *gyrB* phylogenetic tree was inferred with MEGA5.05 (Tamura et al., 2011) using Neighbor-Joining (Saitou and Nei, 1987) and Maximum Likelihood (ML) methods (Felsenstein, 1981). All positions that contained gaps or missing data were eliminated from the final dataset, resulting in 1911 bp positions of *gyrB* sequence. We used 729,383 bp of DNA to represent the conserved core genome found across 25 strains of the *B. subtilis* group, to generate a phylogenomic tree using RAxML (v 7.2.7) (Pfeiffer and Stamatakis, 2010). The phylogenomic tree was then visualized with iTOL (http://itol.embl.de) (Letunic and Bork, 2011).

### BLAST matrix

The BLAST matrix algorithm was used for pairwise comparison of *Bacillus* PGPR strain proteomes, using methods described previously (Friis et al., 2010). The BLAST matrix determines the average percent similarity between proteomes by measuring the ratio of conserved gene families shared between strains to the total number of gene families within each strain. The absolute number of shared and combined gene families for each strain was displayed in matrix output. This matrix shows the number of proteins shared between each proteome.

### Core-genome analysis

The core-genome of 13 *Bacillus* spp. strains was generated using coding and non-coding sequences. Whole genome sequences from these strains were aligned using progressive Mauve (Darling et al., 2004), which identifies and aligns locally collinear blocks (LCBs) in the XMFA format. LCBs from alignments were collected using stripSubsetLCBs (http://gel.ahabs.wisc.edu/mauve/snapshots/), using minimum lengths of 500 bp. All LCBs were concatenated and converted to multifasta format using a perl script. The same protocol was used to obtain all core sequences, with the exception that the minimum lengths of LCBs were 50 bp, instead of 500 bp. The *Bacillus* spp. core genome was obtained from the comparative alignment of all complete *Bacillus* spp. genomes available in the GenBank as of August 2014 (*n* = 81 genomes). The core genome of the *B. subtilis* group was obtained from comparative analysis of 53 whole genomes of *B. subtilis* strains that included 41 genomes obtained from GenBank and 12 PGPR genomes sequenced in this study. *B. amyloliquefaciens* species-level and *B. amyloliquefaciens* subsp. *plantarum*-level core genomes were generated from 32 *B. amyloliquefaciens* and 28 subsp. *plantarum* genomes. Core genomes were exported to the CLC Genomics Workbench (v 4.9) for evaluation of alignments and annotation using the RAST server (Aziz et al., 2008). The list of *Bacillus* spp. strains used for core genome determination is provided in Supplemental Table 1. Additionally, to identify PGPR-specific core genes, raw sequence reads of PGPR strains sequenced in this study were sequentially reference mapped against the genome sequence of non-PGPR strain *B. subtilis* subsp. *subtilis* str. 168 according to methods described previously (Hossain et al., 2013).

### Identification of core genes uniquely present in *B. amyloliquefaciens* subsp. *plantarum* strains

The aligned genome sequences of 32 *B. amyloliquefaciens* strains and 28 *B. amyloliquefaciens* subsp. *plantarum* strains (which were included within the *B. amyloliquefaciens* strains) were analyzed using CLC Genomics Workbench to obtain the respective species- and subsp.-level core genomes. Trimmed sequence reads of subsp. *plantarum* strain AP193 were reference mapped against the subsp. *plantarum* core genome to obtain core genome-specific sequence reads. The parameters of reference mapping were as follows: mismatch cost = 2, insertion cost = 3, deletion cost = 3, length fraction = 0.5, and similarity = 0.8. Sequence reads mapped to the subsp. *plantarum* core genome were then mapped against the species *amyloliquefaciens* core genome to obtain unmapped sequence reads. These unmapped sequence reads, represent the subsp. *plantarum* core genome that is absent in the *amyloliquefaciens* species-level core genome, were assembled *de novo* using CLC Genomics Workbench then the resulting contigs were uploaded to RAST for gene prediction and annotation. Each ORF, exclusively encoded by the *plantarum* core genome, was further confirmed for uniqueness using BLASTn analysis against the genome sequences of 28 *B. amyloliquefaciens* subsp. *plantarum* and four *B. amyloliquefaciens* subsp. *amyloliquefaciens* strains listed in Supplementary Table 1.

### Prediction of secondary metabolite biosynthesis gene clusters in PGPR strain AP193

Secondary metabolite biosynthesis gene clusters for strain AP193 were predicted using the secondary metabolite identification tool antiSMASH (Blin et al., 2013). Primer-walking PCR was used to fill gaps between contigs containing gene clusters encoding secondary metabolite biosynthesis. Gene prediction and annotation were carried out by GeneMark (Lukashin and Borodovsky, 1998) and BLASTx (NCBI), respectively.

### DNA manipulation and plasmid construction for PGPR strain AP193 mutagenesis

Chromosomal DNA was isolated with the E.Z.N.A. Bacterial DNA Isolation Kit (Omega Biotek, Atlanta, GA) and plasmids were isolated with the E.Z.N.A. Plasmids Mini Kit II (Omega Biotek). Primers used in this study are listed in Table 2. Gene deletion constructs were assembled using splicing through overlap extension PCR (Horton et al., 1989). The assembled products were gel purified with Gel/PCR DNA Fragments Extraction Kit (IBI), digested with appropriate restriction enzymes, and cloned into a pNZT1 vector to construct the delivery plasmids for gene replacement.

**Table 2:** **Primers used in this study**.

**Name**	**Sequence (5′ to 3′)**
HindIII*sfpLL*	ATCAAAGCTTATACGCTGCTTCTGCCTGAT
*SfpLR*	CAGATCCGCGATGTGTTCTT
*SfpRL*	AAGAACACATCGCGGATCTGCGGTCCATATATACTCCGT
PstI*sfpRR*	ATCCTGCAGTGGCGGTTATGCTACAATGA
*SfpUp*	CGCTTTAACACACGGACTGA
*SfpDn*	TTTGTAGGAGCGGGAGAAGA
*SfpDL*	AAAGAGAGGAATCGGGACGA
*SfpDR*	TGTTTTGACGGGGCTGAT
HindIII*SrfLL*	ATCCAAGCTTATATGTACGGTCCGTCGGAA
*SrfLR*	GTTCCATTTGCAGCACTTCA
*SrfRL*	TGAAGTGCTGCAAATGGAACACTGGTCAAGCTGGCTGAAC
PstI*SrfRR*	ATCCCTGCAGGGTGCTTCAGCTCAATTCCT
*SrfUp*	GCGAAAGAGCGTCTGTAGAA
*SrfDn*	AGCCGTCATTGTCAGGTCAA
*SrfDL*	TCGGTCACAGGGAAATCTCT
*SrfDR*	CTGCTTGCGGTACTGCTCT
XhoI*DifLL*	TCAACTCGAGGGCGATTCTCGGTTTATCTC
*DifLR*	GATGGAGGATGCCGGTTAC
*DifRL*	GTAACCGGCATCCTCCATCCAAGAACGCTTTCGGGATT
SpeI*DifRR*	ATCCACTAGTGCCATATCAGATACCGCAGA
*DifUp*	TGGCTGATAAGCACCTACGA
*DifDn*	AAATCCGATTACAGGCGAGA
*DifDL*	ATAAGAAACCCGGTTCGGA
*DifDR*	TGGCGTGACGTCTCTCATC

*Restriction digestion sites are underlined within the sequence of the primers*.

### *In vitro* plasmid methylation using cell free extract of *Bacillus amyloliquefaciens* subsp. *plantarum* AP193

To methylate plasmids prior to transformation into *B. amyloliquefaciens* subsp*. plantarum* AP193, the method developed for *Lactobacillus plantarum* was used with minor modifications (Alegre et al., 2004). Cells from a 100 ml overnight culture of strain AP193 (OD_600_ = 1.3 − 1.5) were pelleted by centrifugation (8000 × g), washed with 100 ml of chilled PENP buffer (10 mM potassium phosphate, 10 mM EDTA, 50 mM NaCl and 0.2 mM PMSF, pH 7.0), and then re-suspended to a final volume of 4 ml. Cells were disrupted by performing two bursts (amplitude 50, pulse 3, and watts 25–30) for 5 min each with a pause of 2 min, using a Vibra-Cell sonicator, and cooled with ice to prevent overheating. Cell debris was removed by centrifugation (8000 × g) at 4°C and the extract was collected through decanting. Three milliliter aliquots of extract were mixed with 3 ml of glycerol (100% v/v) and 0.6 ml of bovine serum albumin (1 mg/ml), then stored at −20°C.

The DNA modification assay was performed in a final volume of 100 μl of the following: 53 μl TNE buffer [50 mM Tris (pH 7.5), 50 mM NaCl, 10 mM EDTA], 10 μl S-adenosylmethionine (0.8 mM), 2 μl BSA (5 mg/ml), 25 μl cell free extract derived from strain AP193 and 10 μl plasmid DNA extracted from *E. coli* K12 ER2925 (0.5–1 μg/μl). The mixture was incubated at 37°C for 16 h. Methylated DNA was extracted with a DNA Clean & Concentrator Kit (Zymo Research, CA), then re-suspended in water and stored at −20°C.

### Electrotransformation of *B. amyloliquefaciens* subsp. *plantarum* AP193

For preparation of electrocompetent cells, strain AP193 was grown overnight in TSB, then diluted 100-fold in NCM to inoculate a subculture. The culture was grown at 37°C on a rotary shaker until the OD_600_ reached 0.7. The cell culture was cooled on ice for 15 min and subjected to centrifugation at 8000 × g for 5 min at 4°C. After washing four times with ice cold ETM buffer (0.5 M sorbitol, 0.5 M mannitol, and 10% glycerol), electrocompetent cells were re-suspended in 1/100 volume of the original culture (Zhang et al., 2011). For electroporation, 100 μl of cells were mixed with 100 ng of plasmid DNA in an ice-cold electroporation cuvette (1 mm electrode gap). Cells were exposed to a single 21 kV/cm pulse generated by Gene-Pulser (Bio-Rad Laboratories) with the resistance and capacitance set as 200 Ω and 3 μF, respectively. The cells were immediately diluted into 1 ml of recovery medium (NCM plus 0.38 M mannitol) (Zhang et al., 2011) and shaken gently at 30 or 37°C for 3 h to allow expression of the antibiotic resistance genes. Aliquots of the recovery culture were then spread onto LB agar supplemented with appropriate antibiotics.

### Two-step replacement recombination procedure for the modification of the strain AP193 genome

A two-step replacement recombinationwas performed as previously described, with minor modifications (Zakataeva et al., 2010). To integrate the plasmid into AP193's chromosome, a single crossover between the target gene and the homologous sequence on the plasmid must occur. To do this, AP193 that contained a delivery plasmid with thedeletion construct was first grown in LB broth for 24 h at 37°C (a non-permissive temperature for plasmid replication). Next, the culture was serially diluted, plated onto LB agar plates with erythromycin, and incubated at 37°C. Clones were screened by colony PCR using two sets of primers. Each set of primers anneals sequences specific to one of the homologous fragments and to the chromosomal region just outside of the other homologous fragment (Table 2). If PCR products had a reduced size, relative to the wild-type genotype for either primer set, this indicated successful chromosomal integration of the plasmid. In the second step, clones of the integrant were cultured with aeration in LB at 30°C for 24–48 h to initiate the second single-crossover event, resulting in excision of the plasmid, yielding erythromycin sensitive (EmS) clones with either a parental or a mutant allele on the chromosome. Colony PCR was used to examine the presence of desired mutations by primer sets that flank the deleted sequence (Table 2).

### Construction of strain AP193 mutants defective in secondary metabolite biosynthesis

All mutant strains generated in this study are indicated in Table 1. The disruption of the *dfnD* gene was achieved as follows: DNA fragments corresponding to positions −867 to +247 and +643 to +1570 with respect to the *dfnD* translation initiation site were PCR amplified using AP193 genomic DNA as a template. The two fragments were then assembled by fusion PCR. A frameshift mutation was introduced during fusion to ensure complete disruption of the gene. The deletion construct was digested with XhoI and SpeI, then cloned into pNZT1, yielding pNZ-dif. The plasmid was methylated *in vitro* as described above and introduced into strain AP193 by electroporation. Once introduced into strain AP193, plasmid pNZ-dif generated the isogenic mutant AP193Δ*dfnD* by two-step replacement recombination.

To generate the *sfp* deletion mutant, DNA fragments corresponding to positions −781 to +29, with respect to the *sfp* translation initiation site, and +95 to + 935, with respect to the *sfp* translation termination site, were PCR amplified using AP193 genomic DNA as template, assembled by fusion PCR, digested with HindIII and PstI, and cloned into pNZT1 to construct pNZ-sfp. The plasmid pNZ-sfp was used to generate mutant AP193Δ*sfp* using procedures described above.

The Δ*srfAA* mutant was obtained as follows: DNA fragments corresponding to positions +5375 to +6091 and +6627 to +7366, with respect to the *srfAA* translation initiation site, were PCR-amplified, fused by fusion PCR, digested with HindIII and PstI and cloned into pNZT1 as pNZ-srf. Similarly, a frameshift mutation was introduced during the fusion of the upstream and downstream fragments of the target deletion sequence to ensure complete disruption of the gene. The plasmid pNZ-srf was used to generate mutant AP193Δ*srfAA* using procedures described above.

### *In vitro* antimicrobial activities of PGPR strain AP193 and its mutants against plant pathogens

Plant pathogens *Pseudomonas syringe* pv. tabaci, *Rhizobium radiobacter, Xanthomonas axonopodis* pv. vesicatoria, and *Xanthomonas axonopodis* pv. campestris were grown in TSB until the OD_600_ reached 1.0. The wild type strain AP193, as well as the three isogenic mutants Δ*dfnD*, Δ*sfp*, and Δ*srfAA* developed in this study, were grown at 30°C in TSB for 48 h at 220 rpm. Cultures were then centrifuged at 10,000 × g for 2 min then supernatant was passed through a 0.2 μm nylon filter (VWR, PA). For antibiosis assays, 100 μl of an overnight culture for each plant pathogen was spread onto TSA plates (Thermo Scientific, NY) separately then sterile cork borers (10 mm diameter) were used to bore wells in agar plates. Filtered supernatant of AP193 and its three mutants were separately added to fill wells. Plates were allowed to dry and then incubated at 30°C overnight. Zones of inhibition were measured and compared between mutants and wild-type strain AP193 to determine their antimicrobial activities against plant pathogens.

### LC-MS analysis of bacterial supernatants

Bacterial cultures were grown in 2 ml TSB for 72 hours and then cells were removed by centrifugation at 10,000 × g for 10 min, followed by 0.2 μm filtration of the culture supernatant. Samples were analyzed by direct injection from *m/z* 50 to 1200 on a ultra-high pressure liquid chromatography/QTof-mass spectrometer (Waters Acquity UPLC and Q-Tof Premier, Milford, MA) operated at a spray voltage of 3.03 kv and the source temperature of 100°C. The MS analysis was conducted in negative ion mode with a mobile phase of 95% acetonitrile, 5% water, and 0.1% formic acid.

### *In vivo* antibiosis of strain AP193 and its mutants against a plant pathogen

Rutgers tomato seeds (Park Seed, USA) were sown in Styrofoam trays. Three weeks after planting, seedlings were transplanted into a 4.5 inch square pot with commercial potting substrate (Sunshine mix, Sun Gro Horticulture, Agawam, Maine). Three days after transplanting, plants were sprayed with sterile water or PGPR cell suspensions (10^6^ CFU/ml) that had been washed three times prior to being resuspended in sterile water and normalized at an OD_600_ = 1.0 before being serially diluted. PGPR-inoculated plants were placed into a dew chamber at 100% humidity in the dark for 2 days at 24°C then transferred to the greenhouse. One day later, plants were challenge-inoculated with *X. axonopodis* pv. vesicatoria by spraying approximately 10 ml of a 10^7^ CFU/ml pathogen suspension over each plant. Pathogen-inoculated plants were placed in the dew chamber for 2 days then placed in the greenhouse. Plants were watered once daily. Disease severity ratings and harvest were conducted after 14 days of challenge-inoculation. For disease severity rating, four compound leafs were selected from the bottom of each plant. The disease severity of each of the compound leaves was determined by rating the disease severity of each leaflet and calculating the average rating for the compound leaf. Leaflets were rated using a 0–4 rating scale, where 0 = healthy leaflet, 1 =< 20% necrotic area of the leaflet, 2 = 20–50% necrotic area of the leaflet, 3 = 51–80% necrotic area of the leaflet, 4 = 80–100% necrotic area of the leaflet. In addition, dry shoot and root weights were determined. The experimental design was a randomized complete block with 10 replications per treatment. The experiment was conducted twice.

### Data analysis

All data were analyzed by an analysis of variance (ANOVA), and the treatment means were separated by using Fisher's protected least significant difference (LSD) test at *P* = 0.05 using SAS 9.3 (SAS Institute, Gary, NC, USA).

## Results

### Genome statistics and genetic relatedness of *Bacillus* species

Genome sequences of 12 different PGPR *Bacillus* spp. strains were determined using next-generation sequencing. The summary statistics for each *Bacillus* spp. genome sequences and their assemblies are presented in Table 3. The approximate sizes of *Bacillus* spp. genomes ranged from 2.95 to 4.43 Mbp with an average genome size of 3.93 Mbp, which is similar to the 4.09 Mbp average genome size of complete *B. subtilis* genomes available in GenBank (April, 2015). The percent G+C content of the 12 PGPR *Bacillus* spp. strains ranged from 41.3–46.6%, averaging 45.15%, which is similar to the average percent G+C content of the *B. subtilis* genome sequences available in GenBank (43.72%) (March, 2015). Pairwise average nucleotide identities (ANI), a newly proposed standard for species definition in prokaryotes (Richter and Rosselló-Móra, 2009), were calculated for 13 *Bacillus* PGPR strains to determine their interspecies relatedness among *Bacillus* species. The ANI values for PGPR *Bacillus* spp. strains AB01, AP71, AP79, AP143, AP193, and GB03 against *B. amyloliquefaciens* FZB42 (Chen et al., 2007) were greater than 98% (data not shown), indicating that these PGPR strains are affiliated with the *B. amyloliquefaciens* species. The 98.88% ANI of PGPR strain AP254 to *B. subtilis* subsp*. subtilis* strain 168 suggests that AP254 is affiliated with *B. subtilis* (data not shown). The pairwise ANI comparison of PGPR strains INR7, KCTC 3706T, KCTC 13613T, KCTC 13918T, and KCTC 13622T against each other produce ANI values less than 95% (data not shown) suggests that they are distantly related to each other and represent diverse *Bacillus* species.

**Table 3:** **Summary of draft genomes of ***Bacillus*** species sequenced used in this study**.

**Isolates**	**Number of contigs (>1 kb)**	**Size (total bp in assembly)**	**%G+C**	**NCBI BioProject number**	**NCBI short read archive accession no**.	**Approx. sequence coverage (×)**	**Number of predicted ORFs**
AB01	20	3,903,296	46.4	PRJNA239317	SRX475739	44	3944
AP71	198	4,278,192	45.7	PRJNA239317	SRX475740	15	4531
AP79	47	4,236,770	45.8	PRJNA239317	SRX475741	31	4368
AP143	146	2,956,670	46.6	PRJNA239317	SRX475742	24	3324
AP193	152	4,121,826	46.3	PRJNA239317	SRX475807	37	4159
AP254	59	4,048,419	43.8	PRJNA239317	SRX475808	29	4717
GB03	26	3,849,547	46.5	PRJNA227787	SRX380920	560	3928
INR7	44	3,681,709	41.3	PRJNA227786	SRX447924	750	3857
KCTC 3706T	17	3,935,582	43.7	PRJNA227789	SRX447926	895	4140
KCTC 13613T	23	3,779,696	46.3	PRJNA161489	SRX450083	500	3915
KCTC 13918T	32	4,428,962	45.5	PRJNA227788	SRX450084	1000	4704
KCTC 13622T	33	3,981,302	43.9	PRJNA227791	SRX450086	1000	4299

### Phylogenetic relationship of *Bacillus* strains

A phylogenetic analysis based on *gyrB* gene sequences showed sufficient resolution among *Bacillus* taxa and was consistent with ANI comparisons. Strains AP71, AP79, AP143, AP193, AB01, and GB03 were grouped together with reference strains of *B. amyloliquefaciens* subsp. *plantarum* with high bootstrap support, indicating that they are affiliated with subsp. *plantarum*. The three strains of *B. amyloliquefaciens* subsp. *amyloliquefaciens* DSM7, TA208, and LL3 clustered as a single clade, separated from strains of subsp. *plantarum*, supporting the division of two subspecies in *B. amyloliquefaciens* (Borriss et al., 2011). The placement of strain AP254 with *B. subtilis* subsp. *subtilis* strain 168 as a single clade with strong bootstrap support suggests its affiliation with members of the *B. subtilis* group (Figure 1A). A *gyrB* gene based phylogenetic tree constructed using Maximum Likelihood (ML) methods was also concordant with the phylogeny constructed using Neighbor-Joining methods (data not shown). In addition to the *gyrB*-based phylogeny, we constructed a phylogenomic tree using 729,383 bp of core genome sequences present within the genome of 25 *B. subtilis* group isolates to provide a more refined phylogenetic placement of PGPR strains. The topology and allocation of strains to clades in the *gyrB* phylogeny was similar to the phylogenomic tree (Figure 1B). One notable difference is that the topology of the tree regarding the position of strain *B. siamensis* KCTC13613 differs significantly between the *gyrB*-based tree and the phylogenomic tree, with the *gyrB* based phylogeny placing KCTC13613 in a separate clade whereas the phylogenomic tree included it within a monophyletic group that includes strains of *B. amyloliquefaciens* subsp. *plantarum*.

### BLAST matrix

Genome wide proteome comparisons of 13 PGPR *Bacillus* strains using an all-against-all BLASTp approach demonstrated that PGPR *Bacillus* spp. strains are highly diverse, as indicated by gene family similarity between PGPR *Bacillus* spp. genomes ranging from 32-90% (Supplemental Figure 1). Consistent with the phylogenetic analysis, high similarity was found among strains AP71, AP79, AP193, AB01, GB03, and FZB42, with proteomic similarity ranging from 70 to 90%.

### Core-genome analysis

**Figure 2:**
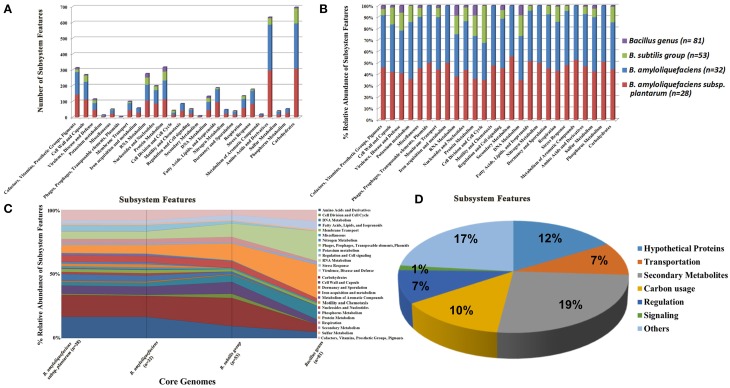
**The distribution of different subsystem categories of four different core genomes specific to genus ***Bacillus*** (***n*** = 81), ***B. subtilis*** subgroup (***n*** = 53), species ***B***. ***amyloliquefaciens*** (***n*** = 32) and subsp. ***plantarum*** (***n*** = 28). (A)** The total counts for genes within different subsystem categories for each of the core genomes. **(B)** The % relative abundance of the genes within different subsystem categories for each of the core genomes. **(C)** Represents the change in % relative abundance of subsystem categories as the number of genomes is reduced after inclusion of additional genomes for core genome prediction. **(D)** Categories of functions encoded by the 73 *B. amyloliquefaciens* subsp. *plantarum*-specific genes present in the *B. amyloliquefaciens* subsp*. plantarum* core genome but absent in the *B. amyloliquefaciens* species-level core genome. The number beside each subgroup of the pie figure represents the number of genes encoding the function.

Analysis of genome sequence alignment using progressive Mauve determined that the core genome of 13 PGPR *Bacillus* spp. strains contains 1,407,980 bp of genomic DNA which encode 1454 ORFs (data not shown). Comparison of core genome sequences of the genus *Bacillus*, subgroup *B. subtilis*, species *B. amyloliquefaciens*, and subspecies *plantarum* demonstrated that as the number of genomes increases, the number of different subsystems within each respective core genome decreases (Figures 2A–C). The highest numbers of subsystems in each of the core genome categories, except for the genus *Bacillus* core genome, was devoted to carbohydrate metabolism. These findings suggest that strains from the genus *Bacillus* use diverse carbon sources. In addition, the core genome for the genus *Bacillus* has more subsystems devoted to RNA, DNA, and protein metabolism compared to carbohydrate metabolism (Figures 2A–C).

The genome alignment from 28 different subsp. *plantarum* strains, including six subsp. *plantarum* strains sequenced in this study, identified 2,550,854 bp of core genome sequence that is predicted to encode 2839 ORFs (Supplemental Table 2). The genome alignment of 32 *B. amyloliquefaciens* strains, including 28 subsp. *plantarum* strains, identified 2,418,042 bp of core genome sequence predicted to encode 2773 ORFs (Supplemental Table 3).

The genome alignment of 53 strains of *B. subtilis* group, including the 12 strains sequenced in this study, identified 578,872 bp of core genome sequence predicted to encode 674 ORFs (Supplemental Table 4). The number of protein coding genes present within the genome of *Bacillus* spp. (~4000) and the low number of ORFs (674) encoded by their core genomes suggests a large amount of genomic plasticity among *Bacillus* genomes that experience frequent gene acquisitions and losses. It was observed that the *B. amyloliquefaciens* core genome was devoid of mobile genetic elements, such as prophages, transposable elements, and plasmids (data not shown). Furthermore, the *B. subtilis* core genome was also devoid of genes or genetic clusters linked with iron acquisition and metabolism, secondary metabolite biosynthesis, signal transduction and phosphorus metabolism (Figures 2A–C).

In this study, the genus *Bacillus* core genome was also determined by analyzing all complete genome sequences from the genus *Bacillus* currently available in GenBank. Our study determined that the genus *Bacillus* contains 194,686 bp of core sequence predicted to encode 201 different ORFs (Supplemental Table 5). The predicted functions present in all *Bacillus* strains are limited to the following subsystem features: cofactor synthesis, vitamin synthesis, prosthetic groups and pigments biogenesis, cell wall and capsule biogenesis, membrane transport, RNA metabolism, nucleoside metabolism, protein metabolism, regulation and cell signaling, DNA metabolism, respiration, amino acids and derivatives, sulfur metabolism, and carbohydrate utilization.

### Comparative analysis of core genes uniquely present in *B. amyloliquefaciens* subsp. *plantarum*

Comparison of PGPR-specific genomes with that of non-PGPR *B. subtilis* subsp. *subtilis* str. 168 did not identify any genes other than essential housekeeping genes that were conserved within the genomes of PGPR strains (data not shown). Comparative analysis of core genomes from 28 *B. amyloliquefaciens* subsp. *plantarum* and 32 *B. amyloliquefaciens* species identified 193,952 bp of sequences that are present within the subsp. *plantarum* core genome but absent in the *B. amyloliquefaciens* core genome. Among these genetic loci there were 73 genes shared by all 28 *plantarum* strains but were not present in any strains of subsp. *amyloliquefaciens* (Supplemental Table 6). The putative functions of these genes includes transportation (7 genes), regulation (7 genes), signaling (1 gene), carbon degradation (10 genes), synthesis of secondary metabolites (19 genes), and hypothetical proteins (12 genes) (Figure 2D). Some of these gene products may be involved in interactions with plants and rhizosphere competence of subsp. *plantarum* strains (e.g., pectin utilization). For instance, genes required for uptake and use of D-galacturonate and D-glucuronate are shared among genomes of *B. amyloliquefaciens* subsp. *plantarum* strains. In addition, genes required for biosynthesis of the polyketides difficidin and macrolactin were consistently found in PGPR subsp. *plantarum* strains, suggesting their relevance in the biocontrol activities of these strains.

### Gene clusters encoding secondary metabolite biosynthesis and natural competency in strain AP193

Due to our observations of beneficial interactions between PGPR strain AP193 and both plant and animal hosts (Ran et al., 2012), we selected this strain for more intensive genome analysis. Assembly of strain AP193 genome sequences *de novo* resulted in 152 contigs larger than 1 kb, with a combined length of 4,121,826 bp. Analysis of AP193 contig sequences, using the antiSMASH secondary metabolite prediction program, suggests that gene clusters were present that are responsible for synthesis of three different polyketides: bacillaene, macrolactin and difficidin. In order to provide complete sequences for these biosynthesis pathways, the gaps between contigs 5 and 6, contigs 33 and 38, as well as contigs 27 and 28 were filled using PCR, followed by DNA sequencing. Each of the gene clusters in AP193 are collinear to their counterparts in *B. amyloliquefaciens* FZB42; a naturally competent plant root-colonizing *B. amyloliquefaciens* isolate with the ability to promote plant growth and suppress plant pathogens (Chen et al., 2007). The percent amino acid identities of the proteins encoded by those clusters were within the range of 98–100% when compared with those of FZB42. Secondary metabolite biosynthesis gene clusters involved in non-ribosomal synthesis of cyclic lipopeptides surfactins, fengycin and bacillomycin D and of the antimicrobial dipeptide bacilysin present in FZB42 were also detected in the AP193 genome. The percent amino acid identities of the AP193 proteins encoded on those clusters to the FZB42 homologs ranged from 98 to 100%. The lack of natural competency of the PGPR strain AP193 prompted us to determine the presence of competence-related genes within this strain. We searched the AP193 genome sequences for the presence of competence related genes found within the genome of FZB42, and observed that all of the genes required for encoding the structural components of the competence system found in strain FZB42 are present within the genome of AP193 with 98 to 100% identity (data not shown); however, genes *comQ, comX*, and *comP* are involved in regulating quorum-sensing in *B. amyloliquefaciens* FZB42 (Chen et al., 2007) were absent within the genome of strain AP193 (data not shown). The absence of *comQ, comX*, and *comP* may be responsible for the lack of natural competency for strain AP193.

### AP193 secondary metabolites inhibit the growth of multiple bacterial plant pathogens *In vitro*

**Figure 3:**
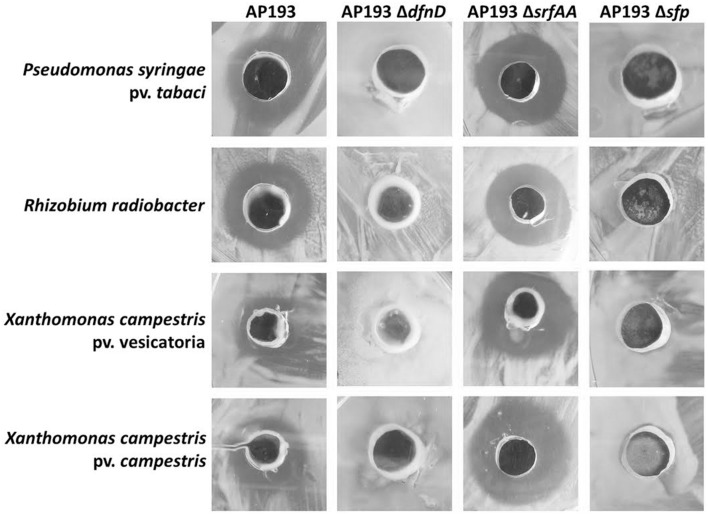
**Antimicrobial activities of ***Bacillus*** sp. AP193 and its mutants Δ***srfAA***, defective in surfactin expression, Δ***dfnD***, defective in difficidin expression, and Δ***sfp***, defective in the expression of multiple secondary metabolites (including difficidin) against plant pathogens ***Pseudomonas syringe*** pv. tabaci, ***Rhizobium radiobacter, Xanthomonas axonopodis*** pv. vesicatoria and ***Xanthomonas axonopodis*** pv. campestris as demonstrated with an agar diffusion assay**.

Antimicrobial activities of strain AP193 and its mutants AP193Δ*dfnD* (deficient in the production of difficidin), AP193Δ*srfAA* (deficient in surfactin production), and AP193Δ*sfp* (unable to produce polyketide or lipopepetide due to a deletion of *sfp* gene encoding 4′-phosphopantetheinyl transferase) were tested against plant pathogens *Pseudomonas syringe* pv. tabaci, *Rhizobium radiobacter, Xanthomonas axonopodis* pv. vesicatoria, and *Xanthomonas axonopodis* pv. campestris. The AP193 wild type strain demonstrated strong antimicrobial activity, whereas the AP193Δ*sfp* mutant was devoid of an inhibitory effect against those plant pathogens (Figure 3), underlining the contribution of lipopeptides and/or polyketides in the bioactivity of AP193. This also indicates that the dipeptide bacilysin, whose synthesis is independent of Sfp, was not involved in antagonistic activity expressed *in vitro*. The AP193Δ*srfAA* mutant conferred antimicrobial activity similar to wild-type to *P. syringe* pv. tabaci, *R. radiobacter, X. axonopodis* pv. vesicatoria, and *X. axonopodis* pv. campestris (Figure 3), suggesting that surfactin has no putative role in the antibacterial activity of AP193 against those plant pathogens under the conditions tested in this study. These findings also demonstrated that surfactin neither influences the antimicrobial compound biosynthesis in AP193 nor does it inhibit antibacterial activities of the antibacterial compounds produced by AP193. Difficidin acts as the major antibiotic in antagonism of AP193 against plant pathogens *P. syringe* pv. tabaci, *R. radiobacter, X. axonopodis* pv. vesicatoria, and *X. axonopodis* pv. campestris as indicated by the lack of the inhibitory effect of the AP193Δ*dfnD* mutant against those plant pathogens (Figure 3).

**Figure 4:**
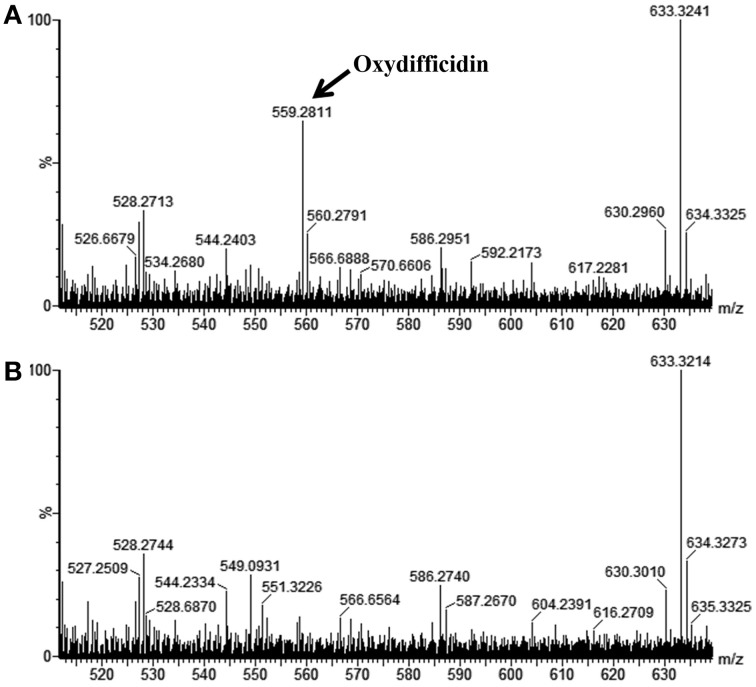
**LC-MS spectra for metabolites from cell-free supernatants of (A) wild-type ***B***. ***amyloliquefaciens*** AP193, and (B) its isogenic ***dfnD*** mutant, when grown in TSB for 72 h**. Note that in negative ion mode that only the deprotonated form of oxydifficidin was detected in bacterial culture supernatants at a *m/z* 559.3.

We further confirmed that the AP193Δ*dfnD* and Δ*sfp* mutants lacked synthesis of difficidin by conducting LC-MS analysis of the cell-free TSB culture supernatants from wild-type AP193 and each of these mutants. As reported previously, only the deprotonated form of oxydifficidin was detectable in bacterial supernatants using MS in the negative mode ([M – H]^−^ = 559.3) (Chen et al., 2006), with a molecular mass of 559.3 detected in supernatants of the wild-type AP193 culture but not observed from the culture of the Δ*dfnD* mutant (Figure 4) or from the Δ*sfp* mutant (data not shown). The Δ*srfAA* mutant exhibited difficidin synthesis as in the wild-type AP193 culture (data not shown). These findings demonstrate the importance of difficidin in the biocontrol activity of subsp. *plantarum* strains against plant pathogens.

### Strain AP193 secondary metabolites control bacterial spot caused by *X. axonopodis* pv. vesicatoria in tomato plants

To determine the role of bioactive compounds produced by strain AP193 in providing protection against plant diseases, the AP193 wild-type strain and its AP193Δ*dfnD*, AP193Δ*sfp*, and AP193Δ*srfAA* mutants were applied to tomato plants several days before those plants were subsequently inoculated with plant pathogen *X. axonopodis* pv. vesicatoria. Both AP193 wild-type and AP193Δ*srfAA* significantly (*P* < 0.05) reduced disease severity of bacterial spot on tomato plants compared to the disease control (Table 4). Additionally, the application of strain AP193 significantly increased the root dry weight of the plants (Table 4). Unlike AP193 wild-type and its AP193Δ*srfAA* mutant, strains AP193Δ*sfp* and AP193Δ*dfnD* neither protected tomato plants from severe bacterial spot caused by *X. axonopodis* pv. vesicatoria nor improved plant growth (Table 4), further supporting the importance of difficidin for plant disease protection. These findings are in agreement with the *in vitro* antibiosis pattern of AP193 wild-type strain and its AP193Δ*dfnD*, AP193Δ*sfp*, and AP193Δ*srfAA* mutants demonstrated against plant pathogen *X. axonopodis* pv. vesicatoria.

**Table 4:** **Effects of plant growth-promoting rhizobacteria (PGPR) strains on severity of bacterial spot disease and plant growth**.

**Strain^a^^b^**	**Disease severity^c^**	**Shoot dry weight (g)**	**Root dry weight (g)**
Disease Control	2.11 a	2.07 bc	0.378 c
AP193	1.30 b	2.18 b	0.453 a
AP193Δs*rfAA*	1.48 b	2.16 b	0.423 abc
AP193Δ*sfp*	2.31 a	2.18 b	0.405 abc
AP193Δ*dif*	2.06 a	2.00 c	0.389 bc
Healthy Control	0.00 c	2.38 a	0.435 ab
LSD	0.35	0.15	0.050

a *The experimental design was a randomized complete block with 10 replications per treatment. The experiment was conducted twice. Values followed by the same letter were not significantly different (P = 0.05) according to Fischer's protected LSD*.

b *One plant was in each replication. Plants were sprayed with PGPR suspension (10^6^ CFU/ml) 1 week after transplanting, and were challenge-inoculated with pathogen solutions (10^7^ CFU/ml) 3 days after inoculating PGPR*.

c *Disease severity ratings and harvest were done 14 days later. For disease severity rating, four compound leafs were selected from the bottom of each plant. The disease severity of each of the compound leaves was determined by rating the disease severity of each leaflet and calculating the average rating for the compound leaf. The leaflet was rated using a 0–4 rating scale, where 0 = healthy leaflet, 1 = < 20% necrotic area of the leaflet, 2 = 20-50% necrotic area of the leaflet, 3 = 51–80% necrotic area of the leaflet, 4 = 80–100% necrotic area of the leaflet, or fully dead leaflet*.

## Discussion

PGPR *Bacillus* spp. strains are used worldwide to improve crop yields and to protect against plant diseases. In this study, 12 PGPR genomes were sequenced, including *B. subtilis, B. pumilus, B. amyloliquefaciens, B. mojavensis, B. siamensis, B. sonorensis*, and *B. tequilensis*. These data were analyzed using ANI, *gyrB*-based phylogenies and core genome-based phylogenies to resolve taxonomic affiliation of *Bacillus* spp. strains. Our findings demonstrate that half of the strains sequenced in this study are affiliated with *B. amyloliquefaciens* subsp. *plantarum*, including strain GB03 that was formerly designated as *B. subtilis*. Previously, *B. siamensis* type strain KCTC 13613T was proposed as a novel species (Sumpavapol et al., 2010), but a *Bacillus* core genome-based phylogenomic analysis (Figure 1) revealed that *B. siamensis* KCTC 13613T is instead affiliated with *B. amyloliquefaciens* subsp. *plantarum*. This finding supports the results of Jeong et al. (2012) that determined the close affiliation of *B. siamensis* type strain KCTC 13613T to *B. amyloliquefaciens* subsp. *plantarum* based on ANI. These findings also support the continued use of core genome-based phylogenomic approaches to provide better phylogenetic resolution than analyses that use a single housekeeping gene (e.g., *gyrB*). Phylogenies based on *gyrB* and core genome sequences demonstrate that *B. amyloliquefaciens* subsp. *plantarum* are highly similar, but comparison of their proteomes demonstrates that they are closely related, yet distinct, and may exert plant growth-promoting activities through different mechanisms.

*B. amyloliquefaciens* subsp. *plantarum* strain AB01 was isolated from the intestine of channel catfish (Ran et al., 2012), but its affiliation with plant-associated strains may suggest transient presence within a fish gastrointestinal tract; however, given that the fish feed is soy-based it is likely that the plant-based diet was also a factor in the growth of this strain within a fish intestine. Similarly, *B. siamensis* type strain KCTC 13613T was found to be closely affiliated with *B. amyloliquefaciens* subsp. *plantarum* and was isolated from salted crab, rather than a plant-associated source. The efficacy of strains AB01, AP193, and other plant-associated strains as probiotics in fish shows the capacity for biocontrol of animal and plant pathogens as well as an overlap in host colonization (Ran et al., 2012).

With rapid advances in sequencing technologies it is now possible to extend genomic analysis beyond individual genomes to analyze core genomes (Medini et al., 2008). In this study, core genomic analyses were conducted on PGPR strains from species affiliated with the *B. subtilis* group. This analysis identified 73 genes exclusively present among all subsp. *plantarum* that are absent in subsp. *amyloliquefaciens* strains. This small number of subsp. *plantarum*-specific genes agrees with a previous report that identified 130 subsp. *plantarum*-specific genes using a limited number of genome sequences from subsp. *plantarum* strains (He et al., 2012). Of these 73 *plantarum*-specific genes identified in this study, many are predicted to be important for plant-associated and soil-associated functions. For example, genes that are required for the use of D-galacturonate and D-glucuronate were found in the pool of *B. amyloliquefaciens* subsp. *plantarum*-specific core genes. This observation is consistent with the absence of these genes in the genome of *B. amyloliquefaciens* subsp. *amyloliquefaciens* DSM7 (Rückert et al., 2011), a strain without any reported PGPR activity. Pectin, a complex polymer found in plant tissues, is broken down to D-glucuronate and D-galacturonate which then serves as a carbon source for bacterial growth (Nemoz et al., 1976). This pectin could potentially serve as a nutrient source for efficient root colonization of PGPR through competitive nutrient uptake. Therefore, the presence of genes that enable D-galacturonate and D-glucuronate utilization could be advantageous for *B. amyloliquefaciens* subsp. *plantarum* for plant growth-promoting activity through efficient root colonization.

Since many of the PGPR strains are from the *B. subtilis* group, the core genome estimation was expanded to include a larger number of *B. subtilis* strains. Increasing the number of *Bacillus subtilis* genomes analyzed to 53 resulted in a 579,166 bp core genome that is predicted to encode 674 ORFs. This smaller number of predicted genes reflects genomic diversity among the *B. subtilis* group. This finding demonstrates that the number of ORFs found in the *B. subtili*s group core genome is close to the number of *B. subtilis* ORFs that are considered as indispensable for growth in complex media (610 ORFs) (http://www.minibacillus.org/project#genes).

To validate a gene's involvement in plant-related processes, it is essential to construct isogenic mutants that are devoid of those genes. Therefore, we deleted genes from PGPR strain AP193 to evaluate the role of secondary metabolite biosynthesis gene clusters in the biological control of plant pathogens. To do this, a methylated shuttle vector pNZT1 (Zakataeva et al., 2010) with gene deletion constructs delivered targeted genetic modifications to AP193, demonstrating the efficacy of *in vitro* methylation of plasmids by cell-free extract in circumventing a restriction system that was presumed to have prevented transformation through electroporation.

Difficidin is a highly unsaturated 22-membered macrocylic polyene lactone phosphate ester with broad-spectrum antibacterial activity (Zimmerman et al., 1987). Difficidin expressed by strain FZB42, together with the dipeptide bacilysin, are antagonistic against *Erwinia amylovora*—the causative agent of fire blight disease in orchard trees (Chen et al., 2009). This study using an isogenic mutant AP193Δ*dfnD* demonstrated for the first time that difficidin solely, not in conjunction with any other polyketides or dipeptides, exerts *in vitro* antibacterial activity against plant pathogens, such as *Pseudomonas syringe* pv. tabaci, *Rhizobium radiobacter, Xanthomonas axonopodis* pv. vesicatoria and *Xanthomonas axonopodis* pv. campestris. We also demonstrated, by using isogenic mutant AP193Δ*dfnD*, that difficidin expression is responsible for control of bacterial spot disease in tomato plants caused by *X. axonopodis* pv. vesicatoria. Taken together, these findings demonstrate that difficidin is the most important strain AP193 secondary metabolite for biological control of plant diseases due to bacterial pathogens. In addition, the construction of the *sfp* gene deletion allowed investigation of multiple secondary metabolites produced by AP193 and their individual contributions to biocontrol activity. The *sfp* deletion mutant lost antagonistic activity against each pathogen that was susceptible to the AP193 wild-type strain. Mutants with the *sfp* deletion are expected to lose the ability to synthesize difficidin in addition to other metabolites. Because the lack of antimicrobial activity of AP193Δ*sfp* is consistent with that of the AP193Δ*dfnD* mutant, this therefore suggests that difficidin is the primary metabolite responsible for *in vitro* inhibition of bacterial pathogens. In contrast, the surfactin mutant retained antimicrobial activity against all plant pathogens tested, demonstrating that surfactin is neither critical for *in vitro* antibiotic activity nor influences the synthesis or secretion of other secondary metabolite biosynthesis in this *Bacillus* spp. strain; however, surfactin may influence plant growth promoting activity since it has been observed that surfactin of *B. subtilis* elicits ISR in plants (Ongena et al., 2007) and is expressed in the plant cells colonized by FZB42 (Fan et al., 2011).

By studying the contributions of genetic loci that are conserved among top-performing PGPR strains we continue to uncover the relative contributions of genes in plant colonization, growth promotion, and/or pathogen biocontrol. In particular, future investigation of genes related to the uptake and use of pectin-derived sugars will help determine the relative importance of these genes for colonization of plants and persistence within this microbiome. Comparative genomic analysis of *Bacillus* spp. PGPR strains has led to a better understanding of gene products and provides a foundation to develop application strategies that result in greater plant growth promotion and biocontrol activity.

### Conflict of interest statement

The authors declare that the research was conducted in the absence of any commercial or financial relationships that could be construed as a potential conflict of interest.

## Supplementary material

Supplemental Table 1 **List of ***Bacillus*** strains used for core genome determination**.

Supplemental Table 2 **Nucleotide and protein sequences of annotated ORFs from the core genome of 28 strains of ***B. amyloliquefaciens*** subsp. ***plantarum*****.

Supplemental Table 3 **Nucleotide and protein sequences of annotated ORFs from the core genome of 32 strains of ***B. amyloliquefaciens*****.

Supplemental Table 4 **Nucleotide and protein sequences of annotated ORFs from the core genome of 53 strains from ***B. subtilis*** group**.

Supplemental Table 5 **Nucleotide and protein sequences of annotated ORFs from the core genome of 81 strains of genus ***Bacillus*****.

Supplemental Table 6 **Nucleotide and protein sequences of annotated 73 ORFs commonly present within the core genome of ***B. amyloliquefaciens*** subsp. ***plantarum*****. This dataset is the basis for the protein categorization presented in Figure 2D.

Supplemental Figure 1 **BLAST matrix of the 13 PGPR ***Bacillus*** spp. strains**. This matrix reveals the pairwise proteomic similarity among the 13 strains. In addition, this matrix presents the absolute number of gene families preserved between any two strains along with the total number of families between them and is used as a basis for the color intensity. The green color represents the % homology between proteomes, and the red color represents % homology within proteomes.
