# Supplementary figures and images for: Deciphering the conserved genetic loci implicated in plant disease control through comparative genomics of Bacillus amyloliquefaciens subsp. plantarum
Source: Front Plant Sci. 2015 Aug 17;6:631. doi: 10.3389/fpls.2015.00631 (PMC4538294; doi:10.3389/fpls.2015.00631)

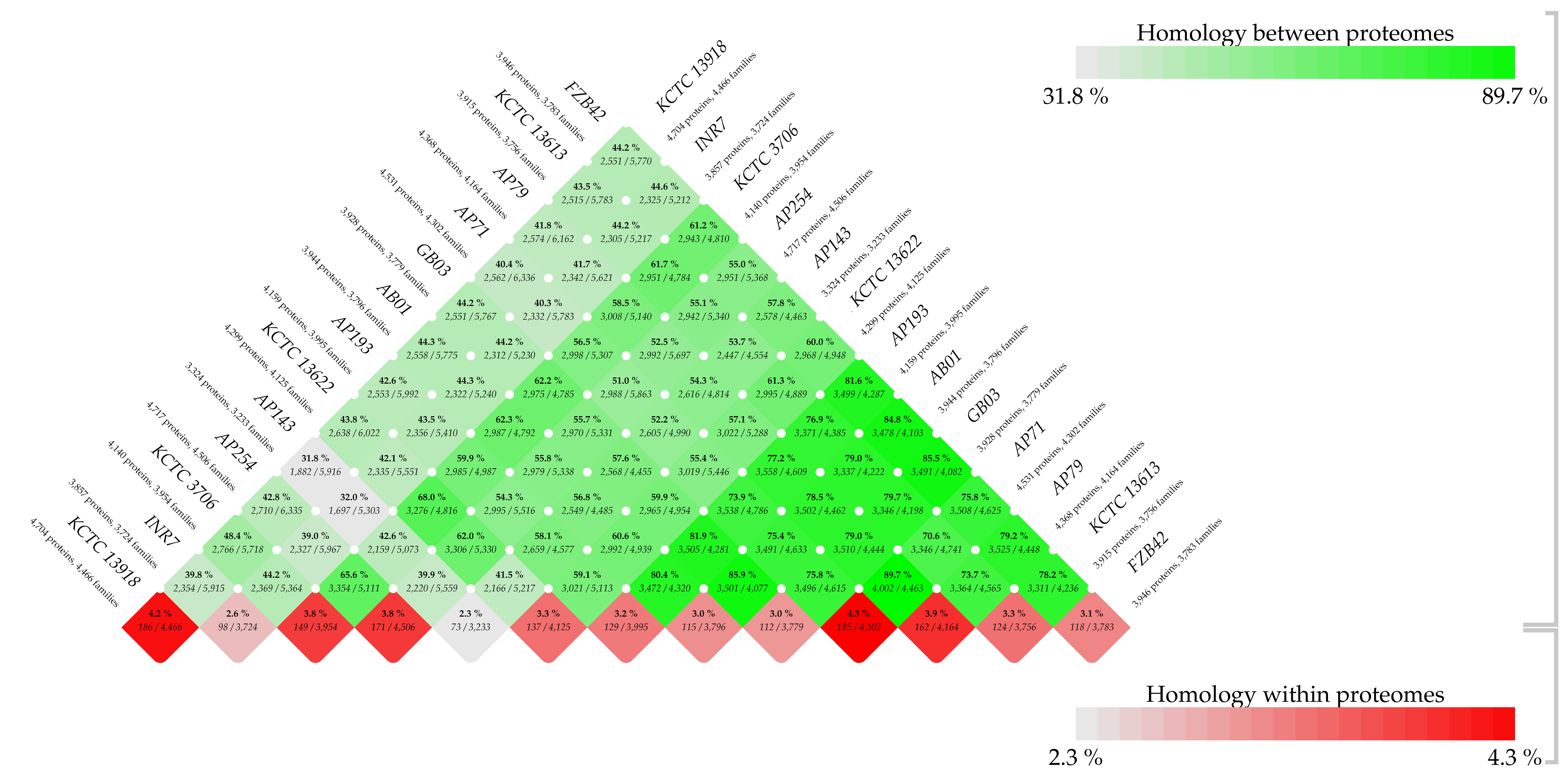

Supplement: Supplemental Figure 1 — BLAST matrix of the 13 PGPR Bacillus spp. strains. This matrix reveals the pairwise proteomic similarity among the 13 strains. In addition, this matrix presents the absolute number of gene families preserved between any two strains along with the total number of families between them and is used as a basis for the color intensity. The green color represents the % homology between proteomes, and the red color represents % homology within proteomes. [file Image1.TIF]
